# Effectiveness of a nurse-led management intervention on systolic blood pressure among type 2 diabetes patients in Uganda: a cluster randomized trial

**DOI:** 10.1186/s40842-024-00173-w

**Published:** 2024-05-20

**Authors:** William Lumu, Silver Bahendeka, Davis Kibirige, Ronald Wesonga, Ronald Kasoma Mutebi

**Affiliations:** 1 Department of Internal Medicine, Mengo Hospital, P.O Box 7161, Kampala, Uganda; 2 Mother Kevin Post Graduate Medical School-Uganda Martyrs University, Kampala, Uganda; 3 Uganda Martyrs Hospital, Lubaga, Kampala, Uganda; 4 School of Statistics and Planning, Makerere University, Kampala, Uganda; 5 Clinical Epidemiology Unit, Makerere University College of Health Sciences, Kampala, Uganda

**Keywords:** Cluster randomized trial, Nurse-led management intervention, Type 2 diabetes, Pooled cohort risk equations, Atherosclerotic cardiovascular risk, Systolic blood pressure, Uganda

## Abstract

**Background:**

Hypertension (HT) is an orchestrator of atherosclerotic cardiovascular disease (ASCVD) in people living with type 2 diabetes (T2D). Control of systolic blood pressure (SBP) and HT as a whole is suboptimal in diabetes, partly due to the scarcity of doctors. While nurse-led interventions are pragmatic and cost-effective in the control of HT in primary health care, their effectiveness on SBP control among patients with T2D in Uganda is scantly known.

**Aim:**

We evaluated the effectiveness of a nurse-led management intervention on SBP among T2D patients with a high ASCVD risk in Uganda.

**Methods:**

A two-armed cluster randomized controlled trial compared the nurse-led management intervention with usual doctor-led care. The intervention involved training nurses to provide structured health education, protocol-based HT/CVD management, 24-h phone calls, and 2-monthly text messages for 6 months. The primary outcome was the mean difference in SBP change among patients with T2D with a high ASCVD risk in the intervention and control groups after 6 months. The secondary outcome was the absolute difference in the number of patients at target for SBP, total cholesterol (TC), fasting blood glucose (FBG), glycated hemoglobin (HbA1C), low-density lipoprotein (LDL), triglycerides (TG), and body mass index (BMI) after the intervention. The study was analyzed according to the intention-to-treat principle. Generalized estimating equations were used to assess intra-cluster effect modifiers. Statistical significance was set at 0.05 for all analyses.

**Results:**

Eight clinics (*n* = 388 patients) were included (intervention 4 clinics; *n* = 192; control 4 clinics; *n* = 196). A nurse-led intervention reduced SBP by -11.21 ± 16.02 mmHg with a mean difference between the groups of -13.75 mmHg (95% CI -16.48 to -11.02, *p* < 0.001). An increase in SBP of 2.54 ± 10.95 mmHg was observed in the control group. Diastolic blood pressure was reduced by -6.80 ± 9.48 mmHg with a mean difference between groups of -7.20 mmHg (95% C1 -8.87 to -5.48, *p* < 0.001). The mean differences in the change in ASCVD score and glycated hemoglobin were -4.73% (95% CI -5.95 to -3.51, *p* = 0.006) and -0.82% (95% CI -1.30 to -0.35, *p* = 0.001), respectively. There were significant absolute differences in the number of patients at target in SBP (*p* = 0.001), DBP (*p* = 0.003), and TC (*p* = 0.008).

**Conclusion:**

A nurse-led management intervention reduces SBP and ASCVD risk among patients with T2D. Such an intervention may be pragmatic in the screening and management of HT/ASCVD in Uganda.

**Trial registration:**

Pan African Clinical Trial Registry, PACTR202001916873358, registered on 6th October 2019.

**Supplementary Information:**

The online version contains supplementary material available at 10.1186/s40842-024-00173-w.

## Background

Atherosclerotic Cardiovascular Disease (ASCVD) causes profound morbidity and mortality in people living with type 2 diabetes (T2D) [1]. Given that the projected rise of diabetes globally is 783 (12.2%) million and in sub-Saharan Africa is 55 million (134%) by the year 2045, there is a potential increase in the burden of ASCVD in this population. Patients with T2D are at a 2-4 fold increased risk for atherosclerotic cardiovascular disease [2], and this risk is heterogeneous [3] due to the occurrence of cardiovascular disease risk factors in different combinations and severity in patients with T2D [4].

Hypertension is one of the major cardiovascular disease risk factors usually screened for in the primary-level setting since it affects 50% of patients with T2D [5]. Diabetes and hypertension are closely related through several pathogenic pathways, including insulin resistance [6]. Both conditions orchestrate CVD in an individual. Therefore, tackling CVD requires effective management of both conditions.

Among patients with T2D, screening and treatment of hypertension is one of the major ways of preventing and managing ASCVD at the Primary level. Relatedly, elevated systolic blood pressure is one of the major contributors to atherosclerotic cardiovascular disease among T2D patients [7], and the benefits of its control have been elucidated. There is evidence that each 10 mmHg reduction in systolic blood pressure significantly reduces all-cause mortality, cardiovascular events, CHD, stroke, albuminuria, and retinopathy, regardless of baseline systolic blood pressure [8, 9].

Despite the available evidence on the benefits of hypertension treatment among patients with T2D, a significant number of patients have suboptimal control, and they are thus potentially at high ASCVD risk.

Suboptimal control of hypertension in Sub-Saharan Africa (SSA), among other factors, is caused by poor access to care and a shortage of physicians [10, 11]. One way of circumventing this problem is to shift the role of screening and treatment of hypertension from Physicians to Non-Physicians when trained and supported.

Currently, there is evidence for nurse-led interventions resulting in better clinical outcomes among patients with hypertension. In several systematic reviews and meta-analyses, nurse-led interventions resulted in better control of hypertension, conformation to treatment guidelines and protocols, better patient satisfaction, and improved access to care [12–19]. Additionally, nurse-led management also leads to better control of diabetes [20] and CVD [21].

While nurse-led interventions are cost-effective with reduced demand on physicians in communicable diseases [22], studies have not evaluated the effectiveness of such interventions on systolic blood pressure and ASCVD among patients with T2D. There is thus paucity of data on the effectiveness of this intervention among patients with T2D in Uganda.

Therefore, the current study was undertaken to assess the effectiveness of a nurse-led management intervention on systolic blood pressure among T2D patients with a high ASCVD risk in Central Uganda.

## Methods

### Trial design

This was a prospective cluster randomized trial with a parallel design that involved eight diabetes clinics (clusters). The clinics were randomized into intervention and control arms with 4 clinics each. The details of the methodology have been stipulated elsewhere [23]. There were no changes to the trial design concerning the eligibility criteria for the cluster and the individual participants after commencement.

### Participants

The study population was comprised of indigenous people born in Uganda and residing in the Central region of the country. The participants were adult patients aged 40 to 79 who were diagnosed and treated for type 2 diabetes in the health facilities selected for this study. Baseline data on the socio-demographic factors, clinical characteristics, and 10-year ASCVD risk of the study participants was obtained. We used the revised Pooled Cohorts Risk Equations [24] to calculate the 10-year ASCVD risk. Participants with a 10-year ASCVD risk of ≥ 7.5% were eligible for the trial. The participants were consecutively selected until the required number was obtained. The study’s health facilities and participants were selected according to the following criteria:

### Eligibility criteria for study health facilities (clusters)

The inclusion criteria for study health facilities were as follows:

I. The study health facility had to run regular outpatient diabetes clinics.
II. The diabetes clinic had to be located in an urban or peri-urban area in the Central Region.
III. The diabetic clinic had to have at least 85 outpatient visits per week to ensure the recruitment of enough participants.
IV. The study clinics had to be more than 6 km apart to minimize contamination due to the sharing of information and other materials between the intervention and control participants.
V. The study health personnel of the enrolled clinics had to be exclusively working for one study health facility to minimize bias.
VI. The diabetes clinics enrolled had to be run by nurses, clinical officers, or medical officers with an updated patient register and a dispensary to ease patient enrollment and distribution of medicine.

### Eligibility criteria for study participants

The inclusion criteria for study participants were as follows:

I. Adult men and women aged 40–79 years with a 10-year ASCVD risk score ≥ 7.5% calculated using the Pooled Cohorts Risks Equations.
II. Patients who were asymptomatic for ASCVD (those without a history of non-fatal myocardial infarction, stroke, heart failure, percutaneous coronary intervention, coronary artery bypass surgery, or current atrial fibrillation).

The following patients were excluded:

I. Pregnant women (The Pooled Cohorts Risks Equations have not been validated in pregnancy).
II. Patients with other comorbidities, such as chronic kidney disease and liver disease.
III. Patients who had skipped two or more of their appointments in the preceding 6 months.

### Study setting and location

**Fig. 1:**
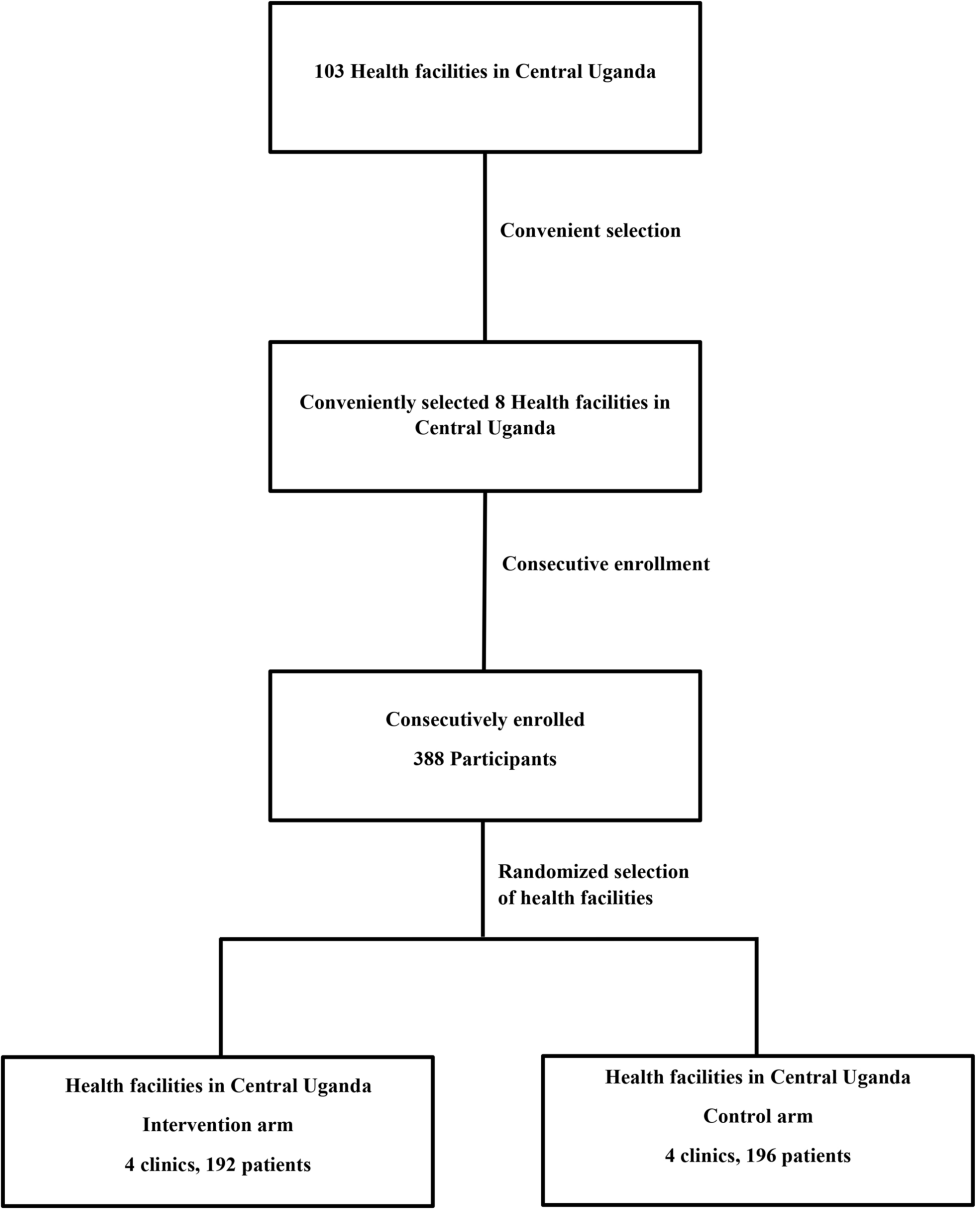
Study flow diagram showing selection of health facilities and participants

The study was conducted in the Central Region of Uganda. This region has 103 Health Center IVs. Seven health facilities were conveniently selected because of their long-term working relationship with Mengo Hospital (these facilities routinely refer T2D patients with cardiovascular disease to Mengo Hospital). The eighth facility selected was Mengo Hospital because it was the study coordinating center. The study health facilities were public and private tertiary hospitals and Health Center IVs. These facilities have updated manual patient registers, and delivery of health care services for the NCDs is done mainly by clinical officers, doctors, and nurses. These facilities run a weekly diabetes clinic where 50–120 patients are treated per clinic day according to the size of the health facility (i.e., smaller facilities tend to have fewer patients than bigger facilities). Details of how the health facilities and participants were selected are shown in Fig. 1.

The clinics (clusters) were randomized and paired according to their location, i.e., urban vs. peri-urban, to balance the arms. Hence, two pairs of urban and peri-urban clinics were created. The details of the randomization process have been provided under the Randomization section.

During clinic days, patients are registered by the nursing assistants or peer leaders. Their anthropometric measurements and blood glucose measurements are done by the triage nurses. Group health education sessions of 15–30 min are conducted by the nurses. Health education sessions are not usually structured but rather are driven by patients’ questions or queries. After health education sessions. Patients are then reviewed and treated by either clinical or medical officers. The prescribed medicines and return dates are recorded by the nurses. The patients are guided to the pharmacy or dispensary to collect their medicine before going home.

### Interventions

The intervention was at both cluster and individual participant levels. In this trial, we evaluated the effect of randomizing clinics to nurse-led management interventions to assess whether this would give similar or better results in terms of systolic blood pressure and ASCVD risk control. All 192 patients in the 4 intervention clinics were subjected to the nurse-led intervention to assess whether they achieved lower systolic blood pressure and ASCVD risk after the 6-month intervention. The nurse-led management intervention was based on Wagner’s Chronic Care Model (CCM) [25].

The intervention involved identifying two nurses from each clinic who were trained for two days at the study head office in Mengo Hospital. We used the curriculum that was specifically developed for this study to train the nurses. The primary objective of the training was to improve the skill and capacity of nurses to screen for and manage hypertension and cardiovascular disease in clinics. The nurses were trained on ASCVD risk screening with the Pooled Cohort Risks Equations, treatment of hypertension and ASCVD, referral of patients for further management, principles of behavioral change, and health promotion.

The nurses were able to give group structured health education on ASCVD prevention and management after the training.

The nurses gave two 30-min health education sessions 3 months apart. The health education topics included the relationship between ASCVD and diabetes, risk factor management, and the benefits of medication adherence. A 15-min question-and-answer session followed each education session. The participants were given an ASCVD leaflet written either in English or the local language (Luganda). The nurses provided the leaflets once at the beginning of the study and encouraged participants to come with them every time they attended the clinic for health education sessions. The main aim of the health education sessions was to obtain an informed, proactive patient to be at the center of the management of his or her condition. Treatment adherence rates and lifestyle change testimonies during health education sessions were used to assess treatment adherence rates. During the initial sessions, the participants elected a leader (Peer Leader) who was trained by nurses to help in registering patients, coordinating health education sessions, and referring critically ill patients to medical facilities from the community. The nurses provided protocol-based hypertension and ASCVD management. A team of three independent physicians coordinated by the principal investigator developed the protocol based on the Uganda Clinical Guidelines on the Management of Hypertension. It was approved by the Ethics Committee.

The nurses were guided by the protocol in the management and provision of medicine (i.e., lipid-lowering agents, anti-hypertensives) and on intra- and inter-facility referrals. The principal investigator reviewed the patients’ files weekly to ensure that nurses’ assessments and prescriptions were appropriate. Where assessments and prescriptions were not appropriate, the principal investigator gave onsite training, and patients were called back to change their prescriptions. The number of patients on appropriate anti-hypertensive and lipid-lowering agents was used by the principal investigator to assess improvement in this capacity. The onsite training and checks galvanized what was taught during the training. This approach underlined the shifting of the role of screening and management of hypertension and ASCVD from a clinical or medical officer to a well-trained nurse with a proactive, educated patient at the center of his or her care.

The intervention provided coaching support to all patients in the intervention arm. The coaching support included individualized telephone calls, text messages, and a 24-h mobile telephone service to address patients’ concerns. The nurses used health education topics to structure the phone calls. Each participant was called for 7 min. Hence, 20 h were used to call 173 patients. During the entire study period, participants were called three times. Call log books were used to record participants and areas discussed. Nurses sent text messages in the local language (Luganda) every 2 months. During enrollment, we inquired about mobile phone ownership, and over 95% of the participants had mobile phones, and the ones without personal mobile phones gave us numbers of their next of kin. Text messages were written on the topics discussed during the health education talks and in the leaflet. A text message log was used to record all the messages. Individualized hypertension and ASCVD management were reinforced by the telephone and text messages at home. Calls and text messages strengthened the nurse-patient relationship, which is a major component of the chronic disease care model.

The doctor- or clinical officer-led care comprised the usual or control arms. Health centers enrolled in the study were health center IVs and higher, where either patients are referred by nurses or refer themselves with symptoms of ASCVD. In these facilities, patients’ ASCVD risk is not assessed, and nurses don’t give structured health education sessions. Screening and treatment of hypertension and ASCVD are done by the clinical officers and medical officers. Management of these conditions is not protocol-based. In these facilities, the management of hypertension and ASCVD varies from facility to facility. Leaflets are not usually given to patients in these health facilities, nor are patients supported with phone calls or text messages. Follow-up of patients in these facilities does not occur at home except at the designated appointment at the health facility.

### Hypothesis

We hypothesized that a nurse-led management intervention reduces systolic blood pressure and ASCVD risk among T2D patients with a high ASCVD risk in the intervention group.

### Specific objective

The trial was conducted to investigate the effect of a nurse-led management intervention on systolic blood pressure among patients with type 2 diabetes with a high ASCVD risk that was implemented in clinics (clusters) in Central Uganda for 6 months.

### Primary and secondary outcomes

The primary outcome measure was the mean difference in change in systolic blood pressure among patients with type 2 diabetes with a high ASCVD risk in intervention and control groups after 6 months. The secondary outcome measure was the absolute difference in the number of patients reaching treatment targets for systolic blood pressure, total cholesterol, fasting blood glucose, glycated hemoglobin, low-density lipoprotein cholesterol, triglycerides, and BMI between the groups after 6 months. We report no changes in trial outcomes after trial commencement.

### Sample size estimation

The sample size was calculated based on a study by Rudd et al. [26] that evaluated the effects of a nurse-led use of algorithm-based prescription. In that study, nurse-led prescription reduced systolic blood pressure by 14.2 mmHg (SD = 17.2) in the intervention arm and 5.7 mmHg (SD = 18.7) in the control arm [26]. Given the fixed number of diabetes clinics (clusters), to adjust for clustering, the formula for a fixed number of equal-sized clusters was used to calculate the number of participants per arm [27].

Therefore, in each arm, we calculated 174 participants, with the entire trial being 348. Allowing for a projected 10% loss, the adjustment was 174/1–0.1 = 193.3 = 194 participants per arm over 6 months. Thus, we selected 388 participants for the study. These were consecutively selected within all the clinics. The participants were randomly assigned, with 192 participants in the intervention arm and 196 participants in the control arm. After randomization of the facilities, the two facilities with the highest number of participants at baseline were randomized into the control arm, giving a slightly higher number. Therefore, we recruited 48 participants per clinic in the intervention arm and 49 participants in each clinic in the control arm.

### Interim analysis

There was no planned interim analysis for this trial.

### Randomization

An independent epidemiologist randomly assigned clinics to either arm in a 1:1 ratio using computer-generated random sequencing with four clinics in each arm, i.e., intervention *n* = 4 and control *n* = 4. The diabetes clinics (clusters) were the units of randomization.

### Allocation and concealment

The independent epidemiologist allocated and assigned health facilities to either group of the study. The allocation was disclosed after baseline measurements in the presence of study nurses. The participants were consecutively selected by the study nurses in all the clinics. About 192 and 196 participants were selected for the intervention and control arms, respectively. Eligible participants consented, and baseline measurements were obtained.

### Implementation (data collection)

After ensuring eligibility, trained study nurses sought written informed consent from the participants who were consecutively enrolled in the clusters. The study nurses were trained to execute the intervention components as well as data collection. Each participant had baseline data collected on socio-demographics, blood pressure, lipids, ASCVD score, fasting blood glucose, body mass index, and glycated hemoglobin, and again after 6 months. The pre- and post-intervention data was collected on the same individuals (cohort design) in both the intervention and control arms and entered into data collection forms. Details of these have been described in the trial protocol [23].

### Blinding (masking)

The participants and study nurses were not blinded during data collection, monitoring, and management.

### Statistical analysis

The study was analyzed based on the intention to treat principle. Missing data was handled as stipulated in our trial protocol, and imputation was performed according to the last observation carried forward for missing data at 6 months [23]. A descriptive analysis of baseline characteristics between groups was performed using student t-test or chi-square test and equivalents as appropriate. The primary outcome of the trial was the mean difference in the change in systolic blood pressure between baseline and 6 months and between the intervention and usual care groups at individual and cluster levels. This was expressed as the mean systolic blood pressure difference between the groups (with 95% CI) and *p*-value. The effect size for the intervention was assessed with the use of Cohen’s d.

We analyzed data using the method stipulated by Campbell et al. In this method, the mean for each cluster was summarized, and these were compared using a paired t-test that was adjusted to account for the intra-cluster correlation by dividing the t-value by the square root of the design effect [28]. Generalized estimating equations were used to assess for intra-cluster effect modifiers such as age, residence, and baseline systolic blood pressure that were pre-specified because they were believed to have a potential impact on the primary outcome.

## Results

### Recruitment, retention, and attrition

**Fig. 2:**
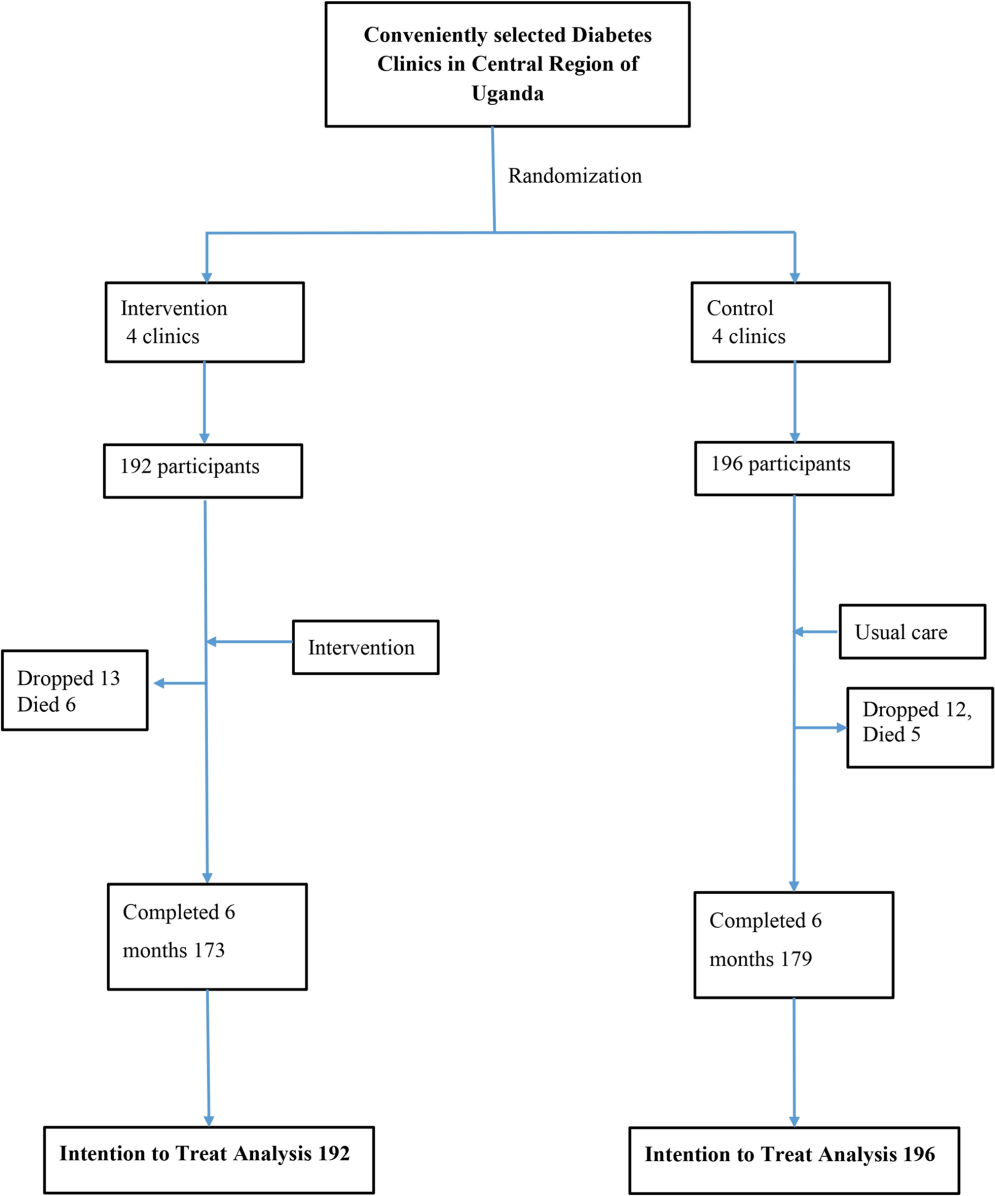
Consort diagram showing assignment and progress of clusters and participants in the study

All clusters (100%) in both groups completed the study. Overall, 352 (90.70%) of the participants completed the trial. In the intervention group, 173 (90.10%, CI 0.44–0.54) participants completed the trial, while 179 (91.30%, CI 0.47–0.57) participants completed it in the control group. Overall, 36 participants did not complete the study, with 19 of them in the intervention group and 17 in the control group. In the intervention group, there were 6 deaths, and 13 participants were lost to follow-up. Five (5) deaths occurred, and 12 participants were lost to follow-up in the control (usual care) group despite attempts by the study coordinator and peer leaders to locate them and find out why they had dropped out. In the intervention group, 5 participants died due to COVID-19 and 1 died due to stroke. In the control group, 2 died due to COVID-19, 2 died due to acute myocardial infarction, and 1 died due to stroke. This is summarized in the CONSORT flow diagram in Fig. 2.

At baseline, more than three-quarters of the participants in both the intervention and control groups were women (78.60% and 77.50%) respectively, and the median age (IQR) of the total population was 58 years (51–63). More participants in the intervention group 98 (51.00%) compared to the control group 65 (33.20%), *p* < 0.001, lived in rural areas. Education level differed between the two groups (*p* = 0.001). Active smoking was low in both arms. Details are shown in Table 1.

**Table 1:** Baseline socio-demographic characteristics across the groups

**Variable**	**Intervention (*n* = 192)**	**Control (*n* = 196)**	***P*-value**
	**n (%)**		
Age *median (IQR)*	57 (50–63)	58 (52–63)	
Gender			
Female	151 (78.60)	152 (77.50)	
Male	41 (21.40)	44 (22.50)	0.794
Residence			
Urban/peri-urban	94 (49.00)	131 (66.80)	
Rural	98 (51.00)	65 (33.20)	< 0.001
Smoking status			
Active (Yes)	1 (0.50)	2 (0.50)	
No smoking (No)	187 (97.40)	190 (97.00)	
Quit	4 (2.10)	4 (2.00)	0.854
Education level			
None	23 (12.00)	4 (2.00)	
Primary	99 (51.60)	107 (54.60)	
Secondary	49 (25.50)	67 (34.20)	
Tertiary	21 (10.90)	18 (9.20)	0.001

*IQR* Interquartile range, frequency, *%* Percentage

At baseline, the mean age ± SD at diabetes diagnosis was 48.90 years (± 9.50) in the intervention group and 50.10 years (± 9.27) in the control group. The median systolic blood pressure was 144.84 mmHg (131.00–158.30) and 137.50 mmHg (127.45–149.50) in the intervention and control groups, respectively. Diastolic blood pressure was higher in the intervention group (87.00 mmHg, IQR: 80.64–95.84) than in the control group (84.85 mmHg, IQR: 78.50–91.00). The median glycated hemoglobin was 8.90% (6.90–11.30) in the intervention group and 8.10% (7.00–10.40) in the control group. The median ASCVD score was 15.09% (9.44–22.43) and 13.21% (8.68–19.44) in the intervention and control groups, respectively. The details are shown in Table 2.

**Table 2:** Median of the baseline characteristics across the groups

**Variable *Median (IQR)***	**Intervention (*n* = 192)**	**Control (*n* = 196)**
Age at DM Diagnosis^a^	48.90 (± 9.50)	50.10 (± 9.27)
DM Duration (IQR)	5 (3.00–10.00)	6 (2.00–11.00)
Systolic BP (mmHg)	144.84 (131.00–158.30)	137.50 (127.45–149.50)
Diastolic BP (mmHg)	87.00 (80.64–95.84)	84.85 (78.50–91.00)
HBA1C (%)	8.90 (6.90–11.30)	8.10 (7.00–10.40)
ASCVD Score (%)	15.09 (9.44–22.43)	13.21 (8.68–19.44)
BMI (kg/m^2^)	28.90 (26.22–33.25)	29.40 (25.65–33.10)
FBG (mmol/l)	8.70 (6.50–11.70)	8.60 (6.70–11.25)
TC (mmol/l)	5.25 (4.55–6.23)	5.41 (4.70–6.40)
LDL (mmol/l)	3.41 (2.61–4.14)	3.58 (2.82–4.29)
HDL (mmol/l)	1.25 (1.07–1.48)	1.27 (1.09–1.58)
TG (mmol/l)	1.89 (1.32–2.53)	1.78 (1.31–2.74)

*n* Frequency, *SD* Standard deviation, *DM* Diabetes mellitus, *BP* Blood pressure, *IQR* Interquartile range, *HbA1c* Glycated hemoglobin, ASCVD Atherosclerotic cardiovascular disease, *BMI* Body mass index, *FBG* Fasting blood glucose, *T.C* Total cholesterol, *LDL-C* Low-density lipoprotein cholesterol, *HDL-C* High-density lipoprotein cholesterol, *TG* Triglycerides

^a^Expressed as mean and SD

### Primary outcome

After 6 months, the mean change in systolic blood pressure was -11.21 ± 16.02 mmHg in the intervention group, while it was + 2.54 ± 10.95 mmHg in the control group. The mean difference in the change in systolic blood pressure between the two groups was -13.75 mmHg (95% CI -16.48 to -11.02, *p* < 0.001). The effect size for the intervention was 0.85.

The effect modifiers for the intervention among the clusters were age (*p* = 0.007), residence (*p* < 0.001), baseline systolic blood pressure (*p* < 0.001), coronary heart disease history (*p* < 0.001), and education level (*p* = 0.002).

### Mean change in other CVD variables

After 6 months, the average change in diastolic blood pressure was -6.80 ± 9.48 mmHg in the intervention group, whereas an average change of + 0.40 ± 5.37 mmHg was observed in the control group. The mean difference in the change in diastolic blood pressure between groups was -7.20 mmHg (95% C1 -8.87 to -5.48, *p* < 0.001), and an effect size of 0.68 was noted.

The mean difference in the change in ASCVD score was -4.73% (95% CI -5.95 to -3.51, *p* = 0.006), glycated hemoglobin -0.82% (95% CI -1.30 to -0.35, *p* = 0.001), fasting blood glucose 0.01 mmol/l (95% CI -0.79 to 0.81, *p* = 0.988), total cholesterol -0.06 mmol/l (95% CI-0.31 to 0.07, *p* = 0.221), LDL-0.08 mmol/l (95% CI -0.26 to 0.10, *p* = 0.387), HDL-0.03 mmol/l (95% CI -0.09 to 0.04, *p* = 0.453), TGs -0.03 mmol/l (95% CI -0.25 to 0.18, *p* = 0.776) and BMI 0.38 kg/m2 (95% CI -0.25 to 1.02, *p* = 0.236). Details of the mean differences between groups and their effective sizes are shown in Table 3.

**Table 3:** Primary and other cardiovascular risk outcomes after 6 months of follow-up

**Variable**	**Mean Change ± SD**		**Mean Difference (95% CI)**	***P*-value**	**ES(d)**
	**Intervention**	**Control**			
**Primary Outcome**					
SBP (mmHg)	-11.21 ± 16.02	2.54 ± 10.95	-13.75 (-16.48 to -11.02)	< 0.001	0.85
**Other CVD risk factors**					
DBP (mmHg)	-6.80 ± 9.48	0.40 ± 5.37	-7.20 (-8.87 to -5.48)	< 0.001	0.68
ASCVD score (%)	-3.30 ± 6.78	1.43 ± 5.37	-4.73 (-5.95 to -3.51)	0.006	0.40
HBA1C (%)	-1.34 ± 2.30	-0.52 ± 2.47	-0.82 (-1.30 to -0.35)	0.001	0.12
FBG (mmol/l)	-0.20 ± 4.33	-0.21 ± 3.66	0.01 (-0.79 to 0.81)	0.988	0.23
TC (mmol/l)	-0.64 ± 1.00	-0.58 ± 0.91	-0.06 (-0.31 to 0.07)	0.221	0.20
LDL-c (mmol/l)	-0.51 ± 0.99	-0.43 ± 0.83	-0.08 (-0.26 to 0.10)	0.387	0.23
HDL-c (mmol/l)	-0.12 ± 0.32	-0.09 ± 0.35	-0.03 (-0.09 to 0.04)	0.453	0.06
TGs (mmol/l)	-0.21 ± 1.20	-0.18 ± 0.94	-0.03 (-0.25 to 0.18)	0.776	0.14
BMI (Kg/M2	-0.02 ± 3.72	-0.40 ± 2.54	0.38 (-0.25 to 1.02)	0.236	0.15

*SD* Standard deviation, *CI* Confidence interval, *ES* Effect size, *d* Coyens, *SBP* Systolic blood pressure, *DBP* Diastolic blood pressure, *ASCVD* Atherosclerotic cardiovascular disease, *HbA1c* Glycated hemoglobin, *FBG* Fasting blood glucose, *TC* Total cholesterol, *LDL* Low-density lipoprotein cholesterol, *HDL* High-density lipoprotein cholesterol, *TG* Triglycerides, *BMI* Body mass index

### Secondary outcome

After 6 months, the absolute difference in the number of patients at target between intervention and control groups was significant for SBP (*p* = 0.001), DBP (*p* = 0.003), and TC (*p* = 0.008). There was no significant absolute difference for FBG (*p* = 0.127) or HBA1 (*p* = 0.495). Details are displayed in Table 4.

**Table 4:** Absolute differences in the number of participants who were at target after 6 months

**Variable**	**Baseline**	**After 6 months**	**Absolute difference**	***P*-value**
	n (%)	n (%)		
**SBP** < 140 mmHg				
Intervention	79 (42.50)	134 (56.30)	55.00	
Control	107 (57.50)	104 (43.70)	-3.00	0.001
**DBP** < 90 mmHg				
Intervention	111 (44.80)	158 (53.70)	47.00	
Control	137 (55.20)	136 (46.30)	-1.00	0.003
**TC** < 5.2 mmol/l				
Intervention	86 (55.80)	130 (54.90)	44.00	
Control	68 (44.20)	107 (45.10)	39.00	0.008
**LDL** < 2.56 mmol/l				
Intervention	45 (53.10)	67 (56.80)	22.00	
Control	38 (46.90)	51 (43.20)	13.00	0.057
**TG**s < 1.685 mmol/l				
Intervention	76 (46.60)	96 (56.10)	20.00	
Control	87 (53.40)	75 (43.90)	-12.00	0.290
**FBG** < 7 mmol/l				
Intervention	62 (53.00)	51 (43.60)	-11.00	
Control	55 (47.00)	66 (56.40)	11.00	0.127
**HbA1**c < 7%				
Intervention	50 (52.10)	77 (51.70)	27.00	
Control	46 (47.90)	72 (48.30)	26.00	0.495
**BMI < 25 kg/**m^2^				
Intervention	37 (48.10)	33 (44.00)	-4.00	
Control	40 (51.90)	42 (56.00)	2.00	0.290

*SBP* Systolic blood pressure, *DBP* Diastolic blood pressure, *TC* Total cholesterol, *LDL* Low-density lipoprotein cholesterol, *FBG* Fasting blood glucose, *TG* Triglycerides, *HbA1c* Glycated hemoglobin, *BMI* Body mass index

## Discussion

This prospective cluster randomized clinical trial showed that a nurse-led intervention resulted in a decrease in systolic blood pressure of -11.21 ± 16.02 mmHg, while an increase in systolic blood pressure of 2.54 ± 10.95 mmHg was observed in the control group. The mean difference between the groups was -13.75 mmHg (95% CI-16.48 to -11.02, *p* < 0.001). Diastolic blood pressure was reduced by -6.80 ± 9.48 mmHg in the intervention group while it increased by 0.40 ± 5.37 mmHg in the control group, with a mean difference in the change of -7.20 mmHg (95% CI-8.87 to -5.48, *p* < 0.001) between the groups. The mean difference in change in 10-year ASCVD risk was -4.73%( 95% CI -5.95 to -3.51, *p* = 0.006) and glycated hemoglobin was -0.82%( 95% CI -1.30 to -0.35, *p* = 0.001) between the groups. We observed a significant difference in the number of patients reaching the target for SBP (*p* = 0.001), DBP (*p* = 0.003), and TC (*p* = 0.008) in the intervention group. There was no significant difference for FBG (*p* = 0.127), HbA1C (*p* = 0.495), or BMI (*p* = 0.290).

The multicomponent intervention in our study, which involved training nurses to give health education and treat hypertension based on a treatment algorithm with readily available anti-hypertensive agents, could explain the higher reduction in systolic blood pressure as medical management is the most effective treatment for hypertension [29]. Additionally, the coaching component of text messaging, phone calls by nurses, and support in the community by the peer leaders could have potentiated good clinical outcomes in our study. There is evidence that coaching interventions result in better clinical outcomes in chronic disease management [30].

Furthermore, our intervention consisted of group counseling that was followed by individual counseling and telephone contacts over the study period. Other than establishing stronger bonds, individual counseling tailors health education needs to a particular person since there is individual variation in cardiovascular risk factors that is not usually addressed by group counseling alone. Our multicomponent intervention embraced the current concept of therapeutic concordance, which is premised on better communication and engaging patients in their treatment decisions, leading to better treatment outcomes and a low incidence of adverse drug reactions, which usually cause non-adherence and dropouts from treatment. In our intervention, we prescribed communication and patient support through periodical text messages and phone calls, which supported the patient in behavioral modification and taking their drugs [31]. Since systolic blood pressure is a major orchestrator of ASCVD [32–35], a reduction in systolic blood pressure also resulted in a reduction in the 10-year ASCVD risk score. We noted an increase in systolic blood pressure in the control group, whose reasons we could not readily ascertain. However, in Uganda, medicine and laboratory tests for treating chronic illnesses such as diabetes, hypertension, and cardiovascular disease are expensive and scarce [36], and our health system, which is modeled to treat communicable diseases, is not yet sufficiently ready to treat non-communicable diseases [37]. Similar findings were observed in a study by Donna L. Mclean et al. in which a community- and nurse-based intervention resulted in a 5.6 mmHg reduction in systolic blood pressure among patients with T2D after 6 months. Differences between the systolic blood pressure reductions in the two studies arise from the difference in the intervention components, i.e., Donna’s study only consisted of empowering patients to take charge of their blood pressure, dietary and physical exercise, documenting their blood pressure, and referring them to their family physicians [29] while our trial consisted of nurse prescription and provision of anti-hypertensive agents that could have led to a higher systolic blood pressure reduction.

In another study done in China to assess the effect of a 6-month community-based intervention on 10-year CHD risk recruited from 2 communities, there was a 5 mmHg reduction in systolic blood pressure (t = 2.01, *p* = 0.47) in the intervention group [38]. The intervention in this study comprised group education and coaching sessions that were similar to our intervention. Nurse prescription and algorithmic HT management in our study could explain the difference in the reduction in systolic blood pressure observed between the two studies. Furthermore, the different study sites, the profile of patients studied, and the CVD risk assessment tools used could underpin the difference in the magnitude of systolic blood pressure reduction. In the same Chinese study [38], there was a reduction in glucose (t = 2.49, *p* = 0.015), total cholesterol (t = 2.44, *p* = 0.017), body mass index (t = 2.58, *p* = 0.011), depression (t = 2.49, *p* = 0.043), and better health-related quality of life (t = 3.36, *p* = 0.01). Our trial showed similar observations in terms of glycemic control, but we didn’t observe any significant differences for total cholesterol, low-density lipoprotein cholesterol, high-density lipoprotein cholesterol, triglycerides, or the body mass index. The reasons for this difference remain unclear.

Our trial showed significant between-group differences in ASCVD risk after intervention. This finding was similar to that demonstrated by Marco Aurellio Lumertz Saffi et al. in a study evaluating the effect of systematic nurse-led individual lifestyle counseling sessions on the reduction of 10-year cardiovascular risk scores in patients with coronary artery disease. In the same study, there was a 13.6% reduction in the risk score in the intervention group compared to an 11% increase in the control group in this secondary prevention study [39]. Despite the use of different CVD risk scores (Pooled Cohorts Risk Equations in our study and Framingham risk score in the Brazilian study), the similarity between the intervention components of these two studies is worth noting. Both studies involved individual counseling and telephone contacts over their study periods. These interventions motivated patients and established stronger bonds with the patients, leading to good clinical outcomes in both studies.

In a Ghanaian study, a nurse-led management strategy among patients with uncontrolled hypertension led to a greater decrease in systolic blood pressure of -20.4 mmHg than health insurance alone -16.8 mmHg, with a net difference in reduction of 3.6 mmHg (*p* = 0.21) at 12 months [10]. A reduction in systolic blood pressure was maintained in each group a year post-intervention. This is a fundamental difference between this study and ours; we didn’t do post-intervention follow-up to examine whether the effect was maintained. In our study, systolic blood pressure increased in the control group, yet there was a reduction of 16 mmHg in the Ghana study. This is attributed to the fact that there was more comprehensive care in the Health Insurance scheme than in our usual care, where patients missed clinic visits, laboratory tests, and medicine due to limitations of funds and frequent stock outs [37]. Whereas our study was conducted among people living with T2D with elevated ASCVD, participants in the Ghanaian study had uncontrolled hypertension (mean BP = 155.9/89.6 mmHg), with CVD risk estimation conducted only in the intervention group. The high mean blood pressure could explain the higher reduction in systolic blood pressure. Patients with higher baseline blood pressure tend to have greater reductions in their blood pressure than those with lower values [40]. The strengths of both studies are that they were conducted in real clinical practice, making it easy for them to be replicated in their respective countries. However, they were both limited by the lack of cost-effective analysis of the intervention before they could be used to influence policy change.

In sub-Saharan East Africa, the prevalence of hypertension has ranged from 26 to 30%, and only 18% of people with hypertension receive treatment. Control is achieved by only 7% of individuals [19]. In patients with type 2 diabetes, control of hypertension is inadequate, with less than 12% achieving optimal targets, hence increasing the risk of premature cardiovascular disease [29]. One of the causes of inadequate control of hypertension in T2D patients is the lack of adequate doctors [19, 37].

One of the pragmatic models that have been implemented to mitigate the shortage of doctors is task-shifting the role of hypertension screening and management to nurses [16].

Our study showed a nurse-led intervention significantly reduces systolic blood pressure and ASCVD risk, underscoring the utility of task shifting as a potential strategy for control of HT and ASCVD among patients with T2D. Task shifting is defined as giving a task normally performed by a physician to a health worker with a different or lower level of education and training [16]. In poor resource settings, if task shifting is not done, there is inadequate care as health systems are challenged by the growing health workforce shortage and imbalance in distribution [16].

A nurse has been at the center of task shifting in chronic disease care like hypertension, diabetes, and cardiovascular disease [19] because she is readily available, spends most time with the patient, and is regularly involved in the provision of lifestyle change health education.

Several community-based programs in Sub-Saharan Africa have shown that nurse-led interventions for the management of hypertension have resulted in a reduction of systolic blood pressure [41–43]. The limitations of these programs compared to our study have been the lack of a comparator and low retention rates of 42%, which could have caused a systematic bias in BP assessment during follow-up and ‘regression’ to the mean. In our trial, the loss to follow-up was only 9.8%, which could probably have been due to medicine incentives, improved patient-nurse relationships, and mobilization through peer leaders. Another limitation of these programs is that they didn’t assess other risk factors such as dyslipidemia and blood glucose except for one in Cameroon, which showed a significant downward trend in fasting blood glucose [44]. Nonetheless, findings from these programs further underpin the utility of nurse-led interventions in the management of hypertension in the community.

In one systematic review and meta-analysis of nurse-led interventions to improve control of blood pressure in people with hypertension, interventions that included stepped treatment algorithm showed greater reduction in systolic blood pressure weighted mean difference -8.2 mmHg 95 CI-11.5 to -4.9, nurse prescribing showed greater reduction in blood pressure(systolic blood pressure -8.9 mmHg 95% C1-12.5 to -5.3 and diastolic blood pressure-4.00 mmHg,95CI -5.3 to-2.7), telephone monitoring showed higher achievement of blood pressure targets(relative risk 1.24,95% CI 1.08 to 1.43), community monitoring yielded a weighted mean difference of systolic blood pressure of -4.8 mmHg 95% CI -7.0 to -2.7 and diastolic -3.5 mmHg 95% CI -4.5 to -2.5) [12]. In our trial, there was a bigger decrease in systolic blood pressure, probably because our intervention consisted of multiple components that were described in the systematic review and meta-analysis. However, it is difficult to discern the effect of each component in our trial.

The above-mentioned evidence of nurse-led interventions in the management of hypertension and other non-communicable diseases was collaborated on by Katende G. et al. in their integrative review of the best available evidence for effective nurse-led care interventions in managing hypertension and other non-communicable diseases [37].

To the best of our knowledge, this is the first clinical trial to investigate the effect of a nurse-led intervention on systolic blood pressure and cardiovascular disease risk among patients with type 2 diabetes in Uganda, and we showed that nurse-led intervention is effective in reducing systolic blood pressure and ASCVD risk among patients with T2D with a high ASCVD risk. Our findings can be used in discussions pertaining to change in the policy of shifting the task of screening and treating hypertension and ASCVD among T2D patients from doctors to nurses if cost-effective analysis shows the financial feasibility of this intervention.

## Limitations of the study

Our study has the following limitations:

i) We did not carry out a cost-effective analysis of the intervention or a post-trial assessment to check for maintenance of the effect of our intervention. ii) We used Pooled Cohorts Risks Equations (PCEs) to estimate ASCVD risk. The PCEs have not been validated in our population. Moreover, it is not a diabetes-specific calculator, though it can accurately estimate the ASCVD risk in this population.

## Conclusion

This prospective cluster randomized trial showed that a nurse-led intervention significantly reduced systolic blood pressure and ASCVD risk among patients with T2D. Our findings underpin the need to shift the role of screening and management of hypertension and ASCVD from doctors to nurses as a pragmatic approach to primary CVD management if a cost-effective analysis of the intervention is feasible.

### Supplementary Information

**Additional file 1.**

## Data Availability

The data sets used and/or analyzed during the current study are available from the corresponding author upon reasonable request.

## Abbreviations

ACE: Angiotensin Converting Enzyme
ACE-D: Angiotensin Converting Enzyme-Drugs
ARB: Angiotensin Receptor Blocker
ASCVD: Atherosclerotic Cardiovascular Disease
BMI: Body Mass Index
BP: Blood Pressure
CCB: Calcium Channel Blockers
CHD: Coronary Heart Disease
CI: Confidence Interval
CKD: Chronic Kidney Disease
DM: Diabetes Mellitus
FBG: Fasting Blood Glucose
HbA1c: Glycated hemoglobin
HDL-c: High-density lipoprotein cholesterol
HT: Hypertension
IQR: Interquartile Range
LDL-c: Low-density lipoprotein cholesterol
N: Frequency
OHA: Oral Hypoglycemic Agent
SD: Standard Deviation
SU: Sulphonyl Urea
TC: Total Cholesterol
TG: Triglycerides

## References

[1] Nahmias A, Stahel P, Xiao C, Lewis GF (2020). Glycemia and atherosclerotic cardiovascular disease: exploring the gap between risk marker and risk factor. Front Cardiovasc Med.

[2] Li MF, Zhao CC, Li TT, Tu YF, Lu JX, Zhang R (2016). The coexistence of carotid and lower extremity atherosclerosis further increases cardio-cerebrovascular risk in type 2 diabetes. Cardiovasc Diabetol.

[3] Bertoluci MC, Rocha VZ (2017). Cardiovascular risk assessment in patients with diabetes. Diabetol Metab Syndr.

[4] Zhao Y (2017). Cardiovascular risk assessment and screening in diabetes. Cardiovasc Endocrinol.

[5] Lastra G, Syed S, Kurukulasuriya LR, Manrique C, Sowers JR (2014). Type 2 diabetes mellitus and hypertension: an update. Endocrinol Metab Clin North Am.

[6] Petrie JR, Guzik TJ, Touyz RM (2018). Diabetes, hypertension, and cardiovascular disease: clinical insights and vascular mechanisms. Can J Cardiol.

[7] Chen G, McAlister FA, Walker RL, Hemmelgarn BR, Campbell NRC (2011). Cardiovascular outcomes in Framingham participants with diabetes. Hypertension.

[8] Emdin CA, Rahimi K, Neal B, Callender T, Perkovic V, Patel A (2015). Blood pressure lowering in type 2 diabetes: a systematic review and meta-analysis. JAMA.

[9] Ettehad D, Emdin CA, Kiran A, Anderson SG, Callender T, Emberson J (2016). Blood pressure lowering for prevention of cardiovascular disease and death: a systematic review and meta-analysis. Lancet (London, England).

[10] Ogedegbe G, Plange-Rhule J, Gyamfi J, Chaplin W, Ntim M, Apusiga K (2018). Health insurance coverage with or without a nurse-led task-shifting strategy for hypertension control: a pragmatic cluster randomized trial in Ghana. PLoS Med.

[11] Kayima J, Nankabirwa J, Sinabulya I, Nakibuuka J, Zhu X, Rahman M, et al. Determinants of hypertension in a young adult Ugandan population in epidemiological transition - the MEPI-CVD survey. BMC Public Health. 2015;15(1). 10.1186/s12889-015-2146-y. 10.1186/s12889-015-2146-y PMC4552375 26315787

[12] Clark CE, Smith LFP, Taylor RS, Campbell JL (2010). Nurse led interventions to improve control of blood pressure in people with hypertension: systematic review and meta-analysis. BMJ.

[13] Horrocks S, Anderson E, Salisbury C (2002). Systematic review of whether nurse practitioners working in primary care can provide equivalent care to doctors. BMJ.

[14] Joshi R, Alim M, Kengne AP, Jan S, Maulik PK, Peiris D (2014). Task shifting for non-communicable disease management in low and middle income countries–a systematic review. PLoS One.

[15] Katende G, Becker K (2016). Nurse-led care interventions for high blood pressure control: Implications for non-communicable disease programs in Uganda. Int J Africa Nurs Sci.

[16] Lekoubou A, Awah P, Fezeu L, Sobngwi E, Kengne AP (2010). Hypertension, diabetes mellitus and task shifting in their management in sub-Saharan Africa. Int J Environ Res Public Health.

[17] Lukewich J, Martin-Misener R, Norful AA, Poitras M-E, Bryant-Lukosius D, Asghari S (2022). Correction: Effectiveness of registered nurses on patient outcomes in primary care: a systematic review. BMC Health Serv Res.

[18] Massimi A, De Vito C, Brufola I, Corsaro A, Marzuillo C, Migliara G (2017). Are community-based nurse-led selfmanagement support interventions effective in chronic patients? Results of a systematic review and meta-analysis. PLoS One.

[19] Spies LA, Bader SG, Opollo JG, Gray J (2018). Nurse-led interventions for hypertension: a scoping review with implications for evidence-based practice. Worldviews Evid Based Nurs.

[20] Hailu FB, Hjortdahl P, Moen A (2018). Nurse-led diabetes self-management education improves clinical parameters in Ethiopia. Front Public Health.

[21] DağistanAkgöz A, Gözüm S (2020). Effectiveness of a nurse-led physical activity intervention to decrease cardiovascular disease risk in middle-aged adults: a pilot randomized controlled study. J Vasc Nurs Off Publ Soc Peripher Vasc Nurs.

[22] Babigumira JB, Castelnuovo B, Lamorde M, Kambugu A, Stergachis A, Easterbrook P (2009). Potential impact of task-shifting on costs of antiretroviral therapy and physician supply in Uganda. BMC Health Serv Res.

[23] Lumu W, Kibirige D, Wesonga R, Bahendeka S (2021). Effect of a nurse-led lifestyle choice and coaching intervention on systolic blood pressure among type 2 diabetic patients with a high atherosclerotic cardiovascular risk: study protocol for a cluster-randomized trial. Trials.

[24] Goff DC, Lloyd-Jones DM, Bennett G, Coady S, D’Agostino RB, Gibbons R (2014). 2013 ACC/AHA guideline on the assessment of cardiovascular risk: a report of the American college of cardiology/American heart association task force on practice guidelines. Circulation.

[25] Wagner EH (1998). Chronic disease management: what will it take to improve care for chronic illness?. Eff Clin Pract.

[26] Rudd P, Houstonmiller N, Kaufman J, Kraemer H, Bandura A, Greenwald G (2004). Nurse management for hypertension. A systems approach. Am J Hypertens.

[27] Hemming K, Girling AJ, Sitch AJ, Marsh J, Lilford RJ (2017). Erratum to: Sample size calculations for cluster randomised controlled trials with a fixed number of clusters. BMC Med Res Methodol.

[28] Campbell MK, Mollison J, Steen N, Grimshaw JM, Eccles M (2000). Analysis of cluster randomized trials in primary care: a practical approach. Fam Pract.

[29] McLean DL, McAlister FA, Johnson JA, King KM, Makowsky MJ, Jones CA (2008). A randomized trial of the effect of community pharmacist and nurse care on improving blood pressure management in patients with diabetes mellitus: Study of Cardiovascular Risk Intervention by Pharmacists-Hypertension (SCRIP-HTN). Arch Intern Med.

[30] Hanssen TA, Nordrehaug JE, Eide GE, Hanestad BR (2009). Does a telephone follow-up intervention for patients discharged with acute myocardial infarction have long-term effects on health-related quality of life? A randomised controlled trial. J Clin Nurs.

[31] Trimarco V, Manzi MV, Izzo R, Mone P, Lembo M, Pacella D (2023). The therapeutic concordance approach reduces adverse drug reactions in patients with resistant hypertension. Front Cardiovasc Med.

[32] Kannel WB, Vasan RS, Levy D (2003). Is the relation of systolic blood pressure to risk of cardiovascular disease continuous and graded, or are there critical values?. Hypertension.

[33] McCarthy CP, Natarajan P (2023). Systolic blood pressure and cardiovascular risk: straightening the evidence. Hypertension.

[34] Wei Y-C, George NI, Chang C-W, Hicks KA (2017). Assessing sex differences in the risk of cardiovascular disease and mortality per increment in systolic blood pressure: a systematic review and meta-analysis of follow-up studies in the United States. PLoS One.

[35] Rao S, Li Y, Nazarzadeh M, Canoy D, Mamouei M, Hassaine A (2023). Systolic blood pressure and cardiovascular risk in patients with diabetes: a prospective cohort study. Hypertension (Dallas, Tex 1979).

[36] Kibirige D, Atuhe D, Kampiire L, Kiggundu DS, Donggo P, Nabbaale J (2017). Access to medicines and diagnostic tests integral in the management of diabetes mellitus and cardiovascular diseases in Uganda: insights from the ACCODAD study. Int J Equity Health.

[37] Katende D, Mutungi G, Baisley K, Biraro S, Ikoona E, Peck R (2015). Readiness of Ugandan health services for the management of outpatients with chronic diseases. Trop Med Int Health.

[38] Huang YJ, Parry M, Zeng Y, Luo Y, Yang J, He GP (2017). Examination of a nurse-led community-based education and coaching intervention for coronary heart disease high-risk individuals in China. Asian Nurs Res (Korean Soc Nurs Sci).

[39] Saffi MAL, Polanczyk CA, Rabelo-Silva ER (2014). Lifestyle interventions reduce cardiovascular risk in patients with coronary artery disease: a randomized clinical trial. Eur J Cardiovasc Nurs J Work Gr Cardiovasc Nurs Eur Soc Cardiol.

[40] Hu H, Zhang J, Wang Y, Tian Z, Liu D, Zhang G (2017). Impact of baseline blood pressure on the magnitude of blood pressure lowering by nifedipine gastrointestinal therapeutic system: refreshing the Wilder’s principle. Drug Des Devel Ther.

[41] Adler AJ, Laar A, Prieto-Merino D, Der RMM, Mangortey D, Dirks R (2019). Can a nurse-led community-based model of hypertension care improve hypertension control in Ghana? Results from the ComHIP cohort study. BMJ Open.

[42] Kengne AP, Awah PK, Fezeu LL, Sobngwi E, Mbanya J-C (2009). Primary health care for hypertension by nurses in rural and urban sub-Saharan Africa. J Clin Hypertens (Greenwich).

[43] Vedanthan R, Kumar A, Kamano JH, Chang H, Raymond S, Too K (2020). Effect of nurse-based management of hypertension in rural western Kenya. Glob Heart.

[44] Kengne AP, Fezeu L, Sobngwi E, Awah PK, Aspray TJ, Unwin NC (2009). Type 2 diabetes management in nurse-led primary healthcare settings in urban and rural Cameroon. Prim Care Diabetes.

