# Supplementary figures and images for: Effectiveness of a nurse-led management intervention on systolic blood pressure among type 2 diabetes patients in Uganda: a cluster randomized trial
Source: Clin Diabetes Endocrinol. 2024 May 20;10:16. doi: 10.1186/s40842-024-00173-w (PMC11103986; doi:10.1186/s40842-024-00173-w)

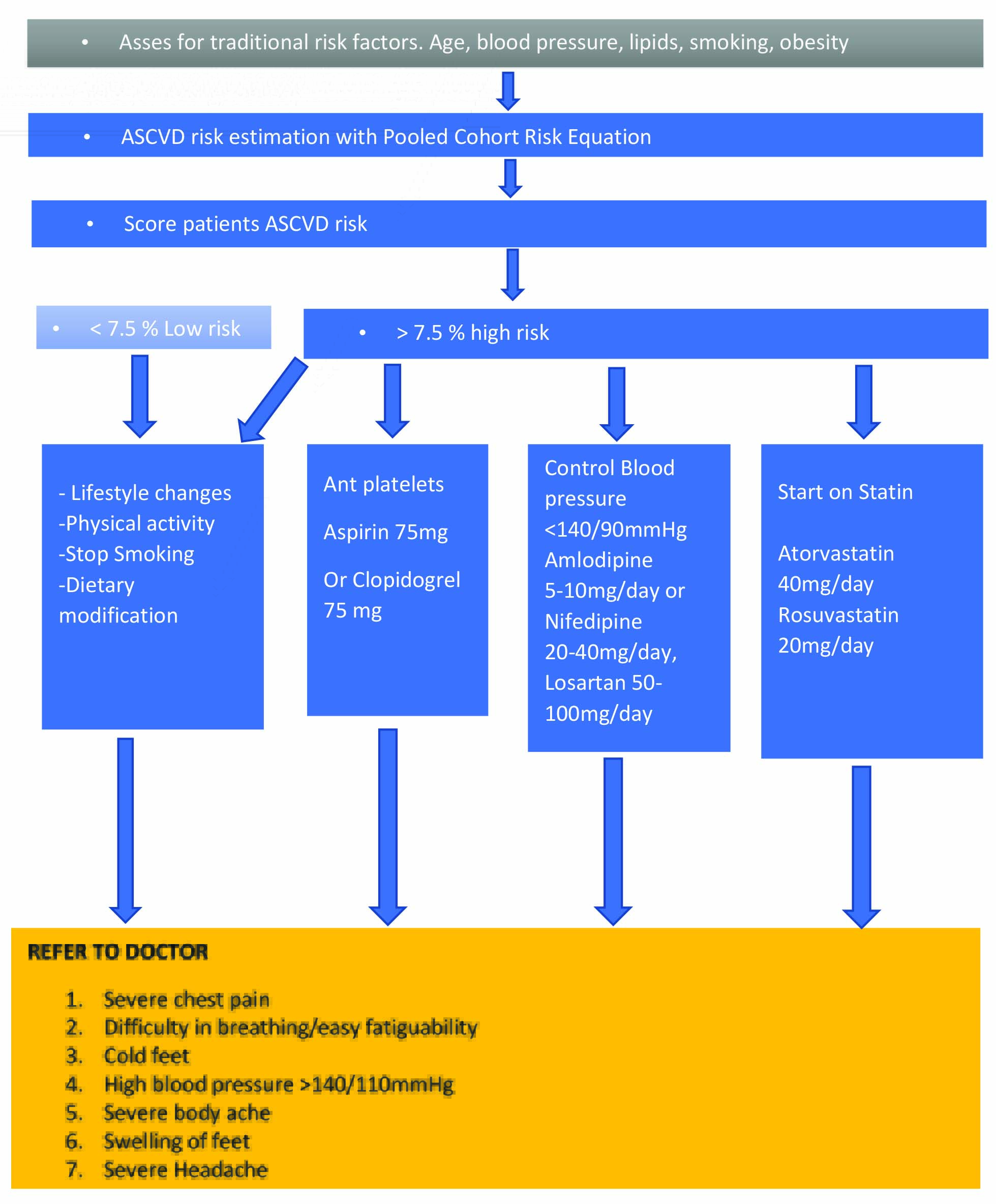

Supplement: Supplementary file 1 — Additional file 1. [file 40842_2024_173_MOESM1_ESM.jpg]
